# Safety and Performance of the Omnipod Hybrid Closed-Loop System in Adults, Adolescents, and Children with Type 1 Diabetes Over 5 Days Under Free-Living Conditions

**DOI:** 10.1089/dia.2019.0286

**Published:** 2020-02-21

**Authors:** Jennifer L. Sherr, Bruce A. Buckingham, Gregory P. Forlenza, Alfonso Galderisi, Laya Ekhlaspour, R. Paul Wadwa, Lori Carria, Liana Hsu, Cari Berget, Thomas A. Peyser, Joon Bok Lee, Jason O'Connor, Bonnie Dumais, Lauren M. Huyett, Jennifer E. Layne, Trang T. Ly

**Affiliations:** ^1^ Division of Pediatric Endocrinology & Diabetes, Department of Pediatrics, Yale University, New Haven, Connecticut.; ^2^ Division of Pediatric Endocrinology, Department of Pediatrics, Stanford University, Stanford, California.; ^3^ Barbara Davis Center, University of Colorado Anschutz Medical Campus, Aurora, Colorado.; ^4^ ModeAGC LLC, Palo Alto, California.; ^5^ Insulet Corporation, Acton, Massachusetts.

**Keywords:** Artificial pancreas, Automated insulin delivery, Closed-loop, Omnipod, Tubeless insulin pump, Pediatrics

## Abstract

***Background:*** The objective of this study was to assess the safety and performance of the Omnipod^®^ personalized model predictive control (MPC) algorithm in adults, adolescents, and children aged ≥6 years with type 1 diabetes (T1D) under free-living conditions using an investigational device.

***Materials and Methods:*** A 96-h hybrid closed-loop (HCL) study was conducted in a supervised hotel/rental home setting following a 7-day outpatient standard therapy (ST) phase. Eligible participants were aged 6–65 years with A1C <10.0% using insulin pump therapy or multiple daily injections. Meals during HCL were unrestricted, with boluses administered per usual routine. There was daily physical activity. The primary endpoints were percentage of time with sensor glucose <70 and ≥250 mg/dL.

***Results:*** Participants were 11 adults, 10 adolescents, and 15 children aged (mean ± standard deviation) 28.8 ± 7.9, 14.3 ± 1.3, and 9.9 ± 1.0 years, respectively. Percentage time ≥250 mg/dL during HCL was 4.5% ± 4.2%, 3.5% ± 5.0%, and 8.6% ± 8.8% per respective age group, a 1.6-, 3.4-, and 2.0-fold reduction compared to ST (*P* = 0.1, *P* = 0.02, and *P* = 0.03). Percentage time <70 mg/dL during HCL was 1.9% ± 1.3%, 2.5% ± 2.0%, and 2.2% ± 1.9%, a statistically significant decrease in adults when compared to ST (*P* = 0.005, *P* = 0.3, and *P* = 0.3). Percentage time 70–180 mg/dL increased during HCL compared to ST, reaching significance for adolescents and children: HCL 73.7% ± 7.5% vs. ST 68.0% ± 15.6% for adults (*P* = 0.08), HCL 79.0% ± 12.6% vs. ST 60.6% ± 13.4% for adolescents (*P* = 0.01), and HCL 69.2% ± 13.5% vs. ST 54.9% ± 12.9% for children (*P* = 0.003).

***Conclusions:*** The Omnipod personalized MPC algorithm was safe and performed well over 5 days and 4 nights of use by a cohort of participants ranging from youth aged ≥6 years to adults with T1D under supervised free-living conditions with challenges, including daily physical activity and unrestricted meals.

## Introduction

Over the past decade, there have been significant advances in the technologies available for the treatment of type 1 diabetes (T1D). Continuous glucose monitors (CGMs) and insulin pumps provide people with T1D information about their glucose levels and trends in real-time, and tools to customize their insulin delivery. Even so, recent data from the United States T1D Exchange (T1DX) Registry indicates that only 17% of youth and 21% of adults meet the American Diabetes Association (ADA) recommended hemoglobin A1c (HbA1c) treatment target.^1^ Automation of insulin delivery with a hybrid closed-loop (HCL) system has the potential to improve glycemic outcomes and reduce the burden of care for people with T1D. One such system, the Omnipod Horizon™ Automated Glucose Control System, is a single-hormone HCL system using a personalized model predictive control (MPC) algorithm under development for commercial applications. This algorithm has previously been shown to be safe in children, adolescents, and adults in an inpatient research environment.^2^ The algorithm was also safe and performed well in adults in a supervised outpatient setting with specific meal and exercise challenges.^3,4^

While assessing safety of HCL systems in controlled settings is an important early step of development, it is crucial to evaluate the safety of an HCL system in free-living conditions that challenge the system with real-world scenarios, such as frequent exercise and unpredictably timed meals and snacks with high fat or high carbohydrate (CHO) content. Free-living trials in a supervised hotel/rental home setting provide the necessary data to bridge between the inpatient setting and home-use studies. In addition, it is essential to evaluate the performance of an HCL system across all age groups to ensure it will perform safely and effectively in people with differing insulin requirements and lifestyles. Insulin dosing may vary widely based on age, residual beta cell function, and insulin sensitivity. For example, children are known to be more sensitive to insulin, oftentimes necessitating a much lower total daily dose (TDD) of insulin compared to adults and adolescents. Adolescents often experience a decline in glycemic control during puberty due to hormonal changes, increased insulin resistance, and behavioral factors such as missed boluses.^5–11^ Several recent studies have evaluated HCL systems in children and adolescents in hotel, camp, and home environments,^12–21^ with results showing improved time in target range. Including all age groups in clinical studies for diabetes technology development will ensure that these systems are better able to support the needs of all people living with T1D.

In addition to testing HCL systems across a wide age spectrum, it is also important to ensure that these systems can be accessed by people with T1D regardless of their prior therapy. Many clinical trials of HCL systems published to date required participants to have prior insulin pump experience for a certain duration (usually 3–6 months), formalizing this as an inclusion criterion for the study.^12–15,22,23^ Yet, this limits the ability to extrapolate study findings to those who use multiple daily injections (MDI) or to assess the transition from MDI to an HCL system.

The objective of this study was to evaluate the safety and performance of the Omnipod personalized MPC algorithm in adults, adolescents, and children aged ≥6 years old with T1D under free-living conditions in a supervised outpatient hotel/rental home setting. An additional aim of this study was to test the feasibility of a direct transition from MDI use to HCL therapy.

## Materials and Methods

### Study design

This single-arm, multicenter study assessed the Omnipod personalized MPC algorithm performance over 96 h in a supervised hotel/rental home setting under free-living conditions, with the HCL period commencing before lunch on study day 1 and ending ∼5 h after breakfast on study day 5. Participants consumed meals and snacks of their own choosing, with no restrictions on nutritional content. The meal bolus amount was calculated based on the CHO content estimated by the participant, or with assistance from the clinical staff, as appropriate, based on age. A correction or reverse bolus at the time of the meal was calculated based on the current sensor glucose and could be overridden at the discretion of the participant. Participants could elect to deliver the meal bolus as an extended bolus, with a portion of the insulin amount delivered initially and the remaining amount infused over a specified duration. Participants were encouraged to engage in physical activity on each full study day, with example activities, including jogging, fitness center, ropes course, scavenger hunt, and trampoline park.

The HCL study phase was preceded by a 7-day outpatient, standard therapy (ST) phase, during which participants managed their diabetes at home per their usual routine using their personal insulin pump or usual MDI regimen. Participants were required to wear the Dexcom G4 505 Share^®^ CGM (Dexcom, San Diego, CA) throughout the ST phase. Partway through the ST phase, clinical staff reviewed insulin and glucose data and made adjustments to the insulin delivery settings as needed, based on their clinical judgment.

### Study participants

Participants included those aged 6 to <65 years, with T1D duration for ≥1 year, HbA1c <10% at screening, and use of any insulin pump or MDI ≥6 months. Potential participants were excluded if they had ≥1 episode of severe hypoglycemia or diabetic ketoacidosis requiring an emergency room visit or hospitalization within the past 6 months, or if they were pregnant or lactating. Each study site obtained Institutional Review Board approvals and written informed consent from adults and the parents or guardians of minors. Written assent was obtained for all minors ≥8 years of age (Clinicaltrials.gov registration NCT03216460).

### Safety and monitoring

Study staff monitored participants' status throughout the HCL study period, with hypoglycemia (capillary blood glucose (BG) <70 mg/dL or symptomatic) or severe hyperglycemia (capillary BG ≥300 mg/dL) treated per standard practice.^24^ Criteria for early discontinuation from the HCL phase of the study included capillary BG ≥300 mg/dL in conjunction with ketones ≥3.0 mmol/L, capillary BG <40 mg/dL for >15 min or <50 mg/dL for >30 min, inability to take oral CHO, loss of consciousness, seizure, or participant request.

### Investigational device

The investigational system used in this study has been described previously.^2–4^ The system uses a modified version of the Omnipod^®^ Insulin Management System (Insulet Corp., Acton, MA) tubeless insulin pump (Pod), a modified Personal Diabetes Manager, the Dexcom G4 505 Share Artificial Pancreas (AP) System, and the Omnipod personalized MPC algorithm running on a Windows 10 tablet configured with the portable AP System.^25^ The system and algorithm were the same across all age cohorts. The default glucose setpoint for this study was 120 mg/dL, although this could be adjusted from 100 to 150 mg/dL in increments of 10 mg/dL for any duration as desired by the participant. The Dexcom G4 was calibrated at least twice daily; however, additional calibrations were performed based on investigator discretion.

Inputs to the investigational Omnipod personalized MPC algorithm included the participant-specific basal rate profile, total daily insulin dose, and the glucose setpoint. Correction factor and insulin to CHO ratio were also entered into the system to be used for calculation of correction boluses and meal boluses. For those on MDI therapy, investigators calculated initial pump settings as per their usual practice, using standard formulas for pump parameters, including determination of the TDD of insulin, and using 500÷TDD for insulin to CHO ratios, 1800÷TDD for correction factors, and setting up basal rates at 40% of the TDD distributed evenly over 24 h. After the first 48 h of HCL, an adaptivity scheme based on standardized formulas was applied to participants' data to determine whether to adjust the participant-specific pump settings. In cases where settings were adapted, participant-specific pump settings were updated manually for the second 48 h of HCL.

### Outcomes

The primary endpoints of this study were safety parameters of percentage of time the sensor glucose was in a hypoglycemic range, defined as <70 mg/dL, and hyperglycemic range, defined as ≥250 mg/dL, during the 96-h HCL study period. Secondary endpoints included mean sensor glucose, percentage time with sensor glucose <54 mg/dL, <60 mg/dL, 70–140 mg/dL, 70–180 mg/dL, >180 mg/dL, and ≥300 mg/dL, and standard deviation and coefficient of variation of sensor glucose values.^26^ The numbers of hypoglycemic (capillary BG values <70 mg/dL) and hyperglycemic events (capillary BG values >300 mg/dL) per participant per day were also analyzed. Sensor glucose data from the at-home ST period were analyzed as a comparison for all glycemic measures. For participants with updates to pump settings during HCL per the adaptivity scheme, exploratory analysis assessed the glycemic outcomes using data from the first 48 h preceding the pump settings update compared to the 48 h following the update. Additional post hoc outcomes included the Glucose Management Indicator (GMI),^27^ daily insulin requirement, and percentage of study time with system operating in HCL.

### Statistical analysis

As the primary endpoint for the study was safety, sample size was not determined by power calculation. Prespecified descriptive statistical analyses were performed for all participants who entered the study per age group, with age groups defined as adults aged ≥18 years, adolescents aged 12 to <18 years, and children aged 6 to <12 years. Results were summarized for the 96-h HCL study period and for the 7-day ST period overall and for the overnight period defined as 23:00 to 07:00. Outcomes were calculated per participant and summarized as mean ± standard deviation or median [interquartile range], unless otherwise indicated. Differences in glycemic outcomes between the ST period and the HCL period were evaluated using the Wilcoxon Signed Rank test for paired observations. Statistical analyses were performed using SAS^®^ 9.3 or later (SAS Institute, Cary, NC).

## Results

A total of 36 participants entered and completed the HCL phase of the trial across 3 study sites (15 at Stanford, 15 at Barbara Davis Center, and 6 at Yale). Table 1 provides participant characteristics by age cohort. Average diabetes duration increased with age across the cohorts, and HbA1c at screening was highest in the adolescent cohort.

**Table 1.** Characteristics of the Study Population

Characteristic	Adults (*N* = 11)	Adolescents (*N* = 10)	Children (*N* = 15)
Age, years (range)	28.8 ± 7.9	14.3 ± 1.3	9.9 ± 1.0
	(19.4–48.5)	(12.6–16.7)	(8.3–11.8)
Female, %	55	40	47
Weight, kg	80.0 ± 21.3	59.4 ± 9.5	37.6 ± 8.5
Diabetes duration, years (range)	14.9 ± 6.9	6.9 ± 3.6	5.2 ± 2.0
	(4.8–26.8)	(2.6–12.0)	(2.5–8.9)
HbA1c, % (range)	7.4 ± 1.2	8.2 ± 1.1	8.0 ± 0.9
	(6.1–9.8)	(6.0–9.9)	(6.5–9.8)
ST, *n* (%)			
Insulin pump	8 (73)	7 (70)	12 (80)
Duration, years (range)	11.4 ± 5.5	7.1 ± 3.5	4.2 ± 2.7
	(4.4–20.7)	(2.7–11.0)	(0.8–8.9)
MDI	3 (27)	3 (30)	3 (20)

Results are mean ± SD unless otherwise indicated.

HbA1c, hemoglobin A1c; MDI, multiple daily injections; SD, standard deviation; ST, standard therapy.

### Glycemic outcomes

The glycemic outcomes for the 96-h HCL period and 7-day ST period for each age group are shown in Table 2. Mean time spent in the target range of 70–180 mg/dL was higher during the 96-h HCL phase compared to the ST phase for all age cohorts, which reached significance for adolescents and children, with an absolute difference of 5.7% for adults, 18.4% for adolescents, and 14.3% for children (*P* = 0.08, *P* = 0.01, and *P* = 0.003). This corresponds to an additional 1.4, 4.4, and 3.4 h in the target range for the respective age cohorts. The difference in time in target range was even more pronounced overnight (23:00–07:00) during HCL compared to ST, with an absolute difference of 8.4% for adults, 23.3% for adolescents, and 20.4% for children (*P* = 0.05, *P* = 0.02, and *P* = 0.0001). This difference equates to an additional time in target range overnight of 0.7 h for adults, 1.9 h for adolescents, and 1.6 h for children.

**Table 2.** Glycemic Outcomes During the 96-h Hybrid Closed-Loop Period and 7-Day Standard Therapy Period

Parameter	Adults (*N* = 11)			Adolescents (*N* = 10)			Children (*N* = 15)		
	HCL	ST	*P*	HCL	ST	*P*	HCL	ST	*P*
Overall									
Mean sensor glucose, mg/dL	150 ± 11	149 ± 26	0.8	144 ± 20	161 ± 25	0.08	156 ± 22	176 ± 28	0.02^*^
Standard deviation, mg/dL	49 ± 9	55 ± 17	0.1	43 ± 11	61 ± 11	0.02^*^	54 ± 10	68 ± 14	0.02^*^
Coefficient of variation, %	33 ± 4	37 ± 7	0.07	29 ± 4	38 ± 6	0.02^*^	35 ± 4	39 ± 3	0.04^*^
Percentage time in glucose range, %									
<54 mg/dL	0.2 ± 0.3	1.2 ± 1.6	0.001^*^	0.4 ± 0.4	0.8 ± 0.8	0.4	0.3 ± 0.4	0.6 ± 0.6	0.2
	0 [0–0.4]	0.6 [0.1–1.7]		0.2 [0.1–0.4]	0.4 [0.1–1.8]		0.2 [0–0.6]	0.5 [0.2–1.0]	
<60 mg/dL	0.4 ± 0.4	2.3 ± 2.6	0.002^*^	0.7 ± 0.8	1.6 ± 1.9	0.4	0.8 ± 0.8	1.1 ± 0.9	0.2
	0.4 [0.1–0.6]	1.4 [0.6–3.0]		0.6 [0.3–1.1]	0.8 [0.5–3.2]		0.6 [0.2–1.7]	0.8 [0.4–1.6]	
<70 mg/dL	1.9 ± 1.3	5.1 ± 4.8	0.005^*^	2.5 ± 2.0	4.4 ± 4.0	0.3	2.2 ± 1.9	2.9 ± 2.4	0.3
	1.7 [0.7–2.6]	3.2 [1.9–7.0]		2.4 [0.8–3.0]	2.8 [1.5–7.5]		1.9 [0.6–2.8]	2.2 [1.5–3.7]	
70–140 mg/dL	49.4 ± 9.2	46.6 ± 16.2	0.4	54.3 ± 15.6	39.9 ± 14.9	0.04^*^	47.1 ± 12.3	34.5 ± 13.5	0.01^*^
70–180 mg/dL	73.7 ± 7.5	68.0 ± 15.6	0.08	79.0 ± 12.6	60.6 ± 13.4	0.01^*^	69.2 ± 13.5	54.9 ± 12.9	0.003^*^
>180 mg/dL	24.5 ± 7.7	26.9 ± 16.7	0.5	18.5 ± 13.5	35.0 ± 16.2	0.03^*^	28.7 ± 14.1	42.2 ± 14.7	0.007^*^
	24.4 [18.1–29.7]	29.8 [10.5–41.2]		12.3 [10.1–24.8]	31.3 [27.8–44.5]		26.1 [17.3–37.0]	45.4 [34.1–50.4]	
≥250 mg/dL	4.5 ± 4.2	7.4 ± 9.6	0.1	3.5 ± 5.0	12.0 ± 6.5	0.02^*^	8.6 ± 8.8	17.5 ± 11.4	0.03^*^
	3.4 [1.5–5.7]	4.7 [1.1–7.6]		0.7 [0.0–8.3]	12.5 [6.7–14.7]		6.3 [3.1–11.5]	16.0 [6.0–24.5]	
≥300 mg/dL	1.1 ± 1.7	2.9 ± 5.8	0.4	0.7 ± 1.0	4.4 ± 3.7	0.02^*^	2.5 ± 3.1	8.3 ± 7.2	0.007^*^
	0.3 [0.0–2.2]	0.0 [0.0–3.6]		0.0 [0.0–2.0]	3.8 [1.6–6.7]		1.5 [0.1–2.8]	6.1 [1.9–13.7]	
Overnight (23:00–07:00)									
Mean sensor glucose, mg/dL	152 ± 32	153 ± 39	0.8	141 ± 27	153 ± 31	0.3	155 ± 29	177 ± 34	0.003^*^
Standard deviation, mg/dL	41 ± 14	54 ± 18	0.01^*^	30 ± 13	54 ± 15	0.01^*^	46 ± 15	61 ± 19	0.03^*^
Coefficient of variation, %	26 ± 6	36 ± 10	0.02^*^	20 ± 5	36 ± 10	0.01^*^	30 ± 6	34 ± 6	0.1
Percentage time in glucose range, %									
<54 mg/dL	0.2 ± 0.5	1.2 ± 2.1	0.2	0.1 ± 0.2	0.7 ± 1.0	0.09	0.1 ± 0.4	0.8 ± 1.1	0.08
	0 [0–0]	0 [0–1.0]		0 [0–0]	0.1 [0–1.4]		0 [0–0]	0.2 [0–1.3]	
<60 mg/dL	0.2 ± 0.6	2.5 ± 4.0	0.07	0.3 ± 0.5	2.2 ± 2.8	0.1	0.3 ± 0.7	1.2 ± 1.4	0.1
	0 [0–0.3]	0.5 [0–2.8]		0 [0–0.5]	0.8 [0–4.0]		0 [0–0]	0.7 [0–2.5]	
<70 mg/dL	0.7 ± 1.1	5.4 ± 7.5	0.05	1.3 ± 1.6	6.5 ± 6.6	0.04^*^	1.0 ± 1.9	2.6 ± 2.5	0.08
	0.3 [0–1.0]	1.9 [0.5–6.9]		0 [0–3.0]	3.6 [1.7–10.3]		0 [0–1.6]	1.9 [0.2–4.4]	
70–140 mg/dL	52.7 ± 28.4	47.3 ± 21.8	0.4	59.6 ± 21.7	41.3 ± 20.1	0.04^*^	52.5 ± 18.4	32.7 ± 16.7	0.0004^*^
70–180 mg/dL	73.9 ± 21.0	65.5 ± 22.7	0.05	85.4 ± 17.9	62.1 ± 15.7	0.02^*^	73.8 ± 19.3	53.4 ± 18.8	0.0001^*^
>180 mg/dL	25.3 ± 21.2	29.1 ± 23.9	0.6	13.3 ± 18.7	31.4 ± 20.5	0.07	25.2 ± 19.7	44.0 ± 20.8	0.0003^*^
	24.3 [14.1–28.1]	23.3 [12.3–38.4]		4.6 [0.0–24.3]	26.4 [19.6–34.9]		19.5 [10.2–36.2]	46.5 [27.7–60.8]	
≥250 mg/dL	6.1 ± 10.9	8.7 ± 14.1	0.3	3.3 ± 7.0	7.5 ± 7.2	0.3	8.6 ± 11.7	16.5 ± 15.0	0.04^*^
	0.5 [0.0–5.5]	4.0 [0.0–11.3]		0.0 [0.0–5.3]	4.6 [1.6–13.9]		3.4 [0.0–17.4]	9.0 [3.8–28.9]	
≥300 mg/dL	1.3 ± 2.8	4.1 ± 8.8	0.2	0.0 ± 0.0	2.9 ± 5.1	0.1	1.8 ± 3.4	7.2 ± 10.3	0.04^*^
	0.0 [0.0–0.3]	0.0 [0.0–6.4]		0.0 [0.0–0.0]	0.0 [0.0–5.6]		0.0 [0.0–3.6]	2.1 [0.0–14.8]	

Results are sensor glucose values, mean ± SD or median [IQR]; SI conversion factor to convert glucose to mmol/L, multiply by 0.0555.

^*^ Statistically significant with *P* < 0.05.

HCL, hybrid closed-loop; IQR, interquartile range.

**FIG. 1.**
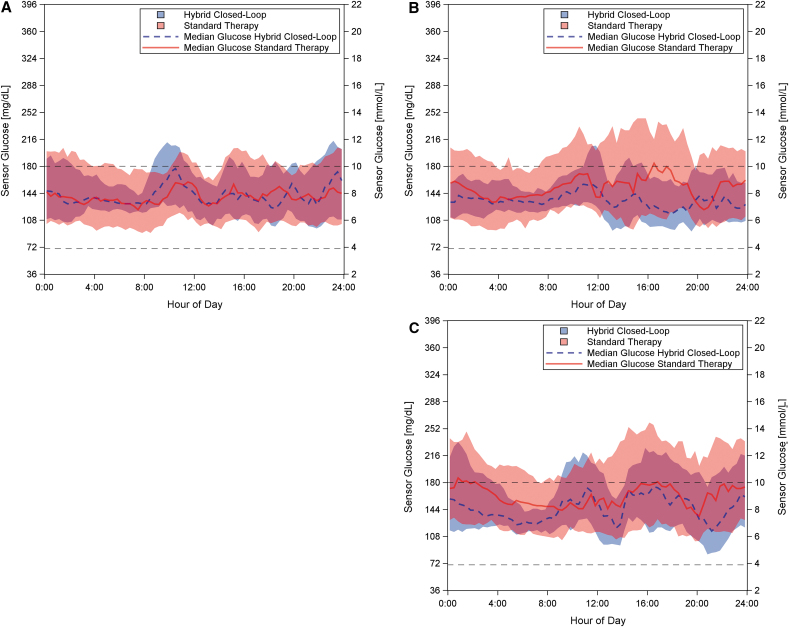
Comparison of the glycemic response for each cohort during the hybrid closed-loop phase compared to the standard therapy phase. Sensor glucose versus time of day for the adult **(A)**, adolescent **(B)**, and pediatric **(C)** participants during 96 h of hybrid closed-loop control (blue dashed line), with data from 1 week of standard therapy shown as comparison (red, solid line). The data are presented as median (line) and interquartile range (shaded area) of sensor glucose per time of day across all participants and days. The target range of 70–180 mg/dL is indicated by black dashed lines.

Sensor glucose profiles during the ST and HCL phases for each study cohort are provided in Figure 1. The adult cohort had a relatively low mean sensor glucose of 149 mg/dL during the ST phase, corresponding to a GMI^27^ of 6.9%; however, they spent an average of 73 min per day in hypoglycemia <70 mg/dL. HCL use led to a significant 2.7-fold decrease in hypoglycemia <70 mg/dL (HCL 1.9% ± 1.3% vs. ST 5.1% ± 4.8%, *P* = 0.005), corresponding to an absolute decrease of 3.2%, or 46 fewer minutes per day. The percentage time in severe hypoglycemia <54 mg/dL also decreased significantly for this cohort (absolute difference of 1%), from an average of 17 min per day during ST to an average of 3 min per day during HCL. In addition, HCL use led to a 1.6-fold reduction in the percentage of time spent in hyperglycemia ≥250 mg/dL (HCL 4.5% ± 4.2% vs. ST 7.4% ± 9.6%, *P* = 0.1), corresponding to an absolute difference of 2.9% (42 fewer minutes per day), although the difference did not reach significance for this cohort. While the mean glucose remained similar during HCL and ST both overall and overnight, the reduction of time spent both in hypo- and hyperglycemia translated to a significant reduction in the coefficient of variation overnight (*P* = 0.02), as is evident in Figure 1A.

Children and adolescents spent more time in hyperglycemia and had a higher mean glucose during the ST phase than adults. In these cohorts, use of HCL had the greatest impact on time spent in hyperglycemia. For children, time spent in hyperglycemia >180 and ≥250 mg/dL was significantly lower during HCL compared to ST, both overall and overnight. Time spent in hyperglycemia >180 mg/dL overnight was reduced from 44.0% ± 20.8% during the ST phase to 25.2% ± 19.7% during HCL (absolute decrease of 18.8%), corresponding to a decrease from 3.5 to 2 h per night (*P* = 0.0003). Furthermore, mean glucose was also significantly lower during HCL compared to ST both for the 24 h period (mean reduction of 20 mg/dL, *P* = 0.02) and overnight (mean reduction of 22 mg/dL, *P* = 0.003).

While the children's cohort experienced marked reduction in hyperglycemia overnight when using HCL, adolescents experienced the greatest reduction in hyperglycemia during the day, as shown in Figure 1B. Hyperglycemia >180 mg/dL was halved (*P* = 0.03) and hyperglycemia ≥250 mg/dL was reduced 3.4-fold (*P* = 0.02) overall during HCL compared to ST, corresponding to an absolute decrease of 16.5% and 8.5%, respectively. This equates to four fewer hours >180 mg/dL and two fewer hours ≥250 mg/dL per day. The mean glucose was lower during HCL compared to ST (mean reduction of 17 mg/dL, *P* = 0.08), although the difference was not found to be significant.

Children and adolescents both saw a significant reduction in coefficient of variation during HCL compared to ST. The mean percentage of time in hypoglycemia was numerically lower during HCL than ST for both adolescents and children, although the difference was not found to be significant. When limited to the overnight period, adolescents had a significant reduction in time spent in hypoglycemia <70 mg/dL, with an absolute decrease of 5.2%, corresponding to 25 fewer minutes per night (HCL 1.3% ± 1.6% vs. ST 6.5% ± 6.6%, *P* = 0.04).

### Physical activity

All participants engaged in daily physical activity for the four full study days, apart from one adult and six children who each had a single day with no recorded physical activity. Children were particularly active during the study, with four children engaging in multiple recorded physical activity sessions on at least one of the study days. Duration per physical activity session was 54 ± 30, 66 ± 27, and 68 ± 51 min for adults, adolescents, and children, respectively. Characteristics regarding intensity of exercise sessions and strategies used to manage physical activity, including use of a raised glucose setpoint or ingestion of supplemental CHOs before exercise, are provided in Table 3.

**Table 3.** Characteristics of Physical Activity with Free-Living Conditions During Hybrid Closed-Loop

	Adults (*N* = 11)	Adolescents (*N* = 10)	Children (*N* = 15)
Exercise sessions, *n*	43	40	61
Exercise duration per session, min	54 ± 30	66 ± 27	68 ± 51
Exercise intensity per session,^a^ %			
Low	49	40	62
Moderate	39	35	30
High	12	25	8
Raised glucose setpoint before exercise, %	37	35	43
Supplemental CHO consumed before exercise (90 min period), %^b^	28	30	26

Data are mean ± SD unless otherwise indicated.

^a^ Evaluated prospectively based on judgment of participant and clinical staff.

^b^ Defined as CHO consumed without a corresponding insulin bolus.

CHO, carbohydrate.

### Meals

Meals and snacks were chosen by participants and were not restricted. This was reflected in the average daily CHO consumption estimates of 186 ± 49, 199 ± 32, and 214 ± 52 g in adults, adolescents, and children, respectively (Table 4). Approximately 20% of meals in adults and adolescents and 30% of meals in children were classified as being high-CHO meals with ≥75 g of CHO. Furthermore, 21%, 30%, and 68% of meals in adults, adolescents, and children, respectively, were defined as high-fat meals with ≥30 g of fat for adults and adolescents and ≥15 g of fat for children.

**Table 4.** Characteristics of Unrestricted Meals with Free-Living Conditions During Hybrid Closed-Loop

	Adults (*N* = 11)	Adolescents (*N* = 10)	Children (*N* = 15)
CHO content			
Total meals, *n*^a^	128	107	179
Mean per meal, g^a^	53 ± 24	57 ± 27	59 ± 30
Min per meal, g^a^	8	5	0
Max per meal, g^a^	154	150	186
Mean per person per 24 h, g^b^	186 ± 49	199 ± 32	214 ± 52
Percentage of high-CHO meals,^c^ %	17	21	27
Fat content			
Total meals with fat content recorded, n^a^	126	79	179
Mean per meal, g^a^	20 ± 17	27 ± 24	25 ± 19
Min per meal, g^a^	1	3	3
Max per meal, g^a^	120	130	105
Mean per person per 24 h, g^a^	57 ± 13	53 ± 20	74 ± 37
Percentage of high-fat meals,^d^ %	21	30	68
Participants with ≥1 high fat meal,^d^*n*	10	10	15
Participants with ≥1 extended bolus, *n*	7	9	7

Data are mean ± SD unless otherwise indicated.

^a^ Included only meals designated as breakfast, lunch, or dinner. Contents for multiple recorded meals of the same type per day were summed.

^b^ Included all recorded CHO including snacks but did not include CHO consumed for hypoglycemia treatments.

^c^ Defined as ≥75 g CHO.

^d^ Defined as ≥30 g fat for adults and adolescents and ≥15 g fat for children.

### Insulin delivery during HCL

Insulin requirements varied widely among the overall study population, with TDD in the ST phase as low as 16 U/day for one participant in the cohort of children to as high as 98 U/day for a participant in the cohort of adults. For adults and adolescents, the TDD tended to decrease during HCL compared to ST as seen in Table 5. This held true regardless of whether modality of insulin delivery before study entry was with a pump or MDI. The same trend was observed for children who used MDI for ST; however, TDD was similar during HCL and ST for children who used an insulin pump for their usual care.

**Table 5.** Insulin Use During the 96-h Hybrid Closed-Loop Period and the 7-Day Standard Therapy Period

TDD of insulin	Adults		Adolescents		Children	
	HCL	ST	HCL	ST	HCL	ST
Overall, *n*	11		10		15	
Units/day	46 ± 16	54 ± 25	44 ± 12	60 ± 18	30 ± 11	32 ± 9
Units/kg/day	0.58 ± 0.19	0.67 ± 0.24	0.73 ± 0.18	1.01 ± 0.24	0.78 ± 0.19	0.84 ± 0.13
Insulin dose based on prior insulin delivery modality						
Prior pump users, *n*	8		7		12	
Units/day	44 ± 16	54 ± 28	49 ± 9	66 ± 17	30 ± 12	30 ± 9
Units/kg/day	0.53 ± 0.09	0.62 ± 0.17	0.82 ± 0.14	1.10 ± 0.21	0.82 ± 0.19	0.83 ± 0.13
Prior MDI users, *n*	3		3		3	
Units/day	50 ± 19	56 ± 22	31 ± 10	45 ± 8	29 ± 6	39 ± 4
Units/kg/day	0.71 ± 0.33	0.80 ± 0.38	0.53 ± 0.06	0.79 ± 0.16	0.65 ± 0.05	0.88 ± 0.16

Data are mean ± SD unless otherwise indicated. HCL insulin dose is averaged over the entire 96-h HCL period.

TDD, total daily dose.

### Parameter adjustments

Adjustments to the participant-specific pump settings used by the algorithm occurred after the first 48 h of the HCL phase based on controller-determined basal rate requirements for 9% (*n* = 1) of the adult participants, 60% (*n* = 6) of the adolescent participants, and 33% (*n* = 5) of the children's cohort. Glycemic outcomes for the first 48 h (before adjustment) and second 48 h (after adjustment) of HCL for these participants are shown in the Supplementary Table S1.

### Safety outcomes

There were no serious adverse events, and the full HCL period was completed for all participants with no instances of the early discontinuation criteria being met. In the approximate 1056, 960, and 1440 patient-hours of HCL use for adults, adolescents, and children, respectively, there were 6, 3, and 12 hyperglycemic events involving capillary BG values >300 mg/dL in 5 adults, 3 adolescents, and 7 children. This is equivalent to 0.14, 0.075, and 0.20 events per participant per 24 h of use overall (median 0 events per participant per 24 h).

During the 96-h HCL period, there were 44, 43, and 62 hypoglycemic events involving capillary BG values <70 mg/dL in 11 adults, 10 adolescents, and 14 children. This is equivalent to 1.0 ± 0.5, 1.1 ± 0.5, and 1.0 ± 0.8 events per participant per 24 h of use overall (median 1.0, 1.0, and 0.8 events per participant per 24 h, respectively). There were four, three, and seven hypoglycemic events occurring overnight (23:00–07:00) in four adults, two adolescents, and three children, equivalent to an average of 0.1 events per participant per night of use (median 0 events per participant per night of use). Three of the overnight events in children occurred in the first hour of the overnight period, with two occurring within 1 h of a bolus provided for food. Hypoglycemic events occurring within 4 h of the end of recorded physical activity accounted for 30% of total events for adults, 65% for adolescents, and 27% for children.

### Percentage time in HCL

The mean percentage of the total HCL study period spent with the system running in closed-loop was 97.5% ± 1.8% (range: 94.4%–99.6%), 98.5% ± 1.3% (range: 95.7%–99.7%), and 98.3% ± 1.6% (range: 94.0%–99.7%) for adults, adolescents, and children, respectively.

## Discussion

This multicenter feasibility study demonstrated that the Omnipod personalized MPC algorithm was safe and performed well over 96 h of use by a diverse population of insulin pump and MDI users, from children to adults with differing insulin requirements, under free-living conditions in a supervised outpatient setting. Regardless of age, the mean percentage time in target range during HCL was higher than that during ST, with less hyperglycemia. Furthermore, this was achieved with a reduction of time in hypoglycemia.

Importantly, this study was among the first to enroll both insulin pump and MDI users. The successful transition of those on MDI to HCL in this feasibility trial provides the first evidence to support the option of quickly transitioning MDI patients to this HCL system when it becomes commercially available. Indeed, the MDI users had similar time in target range and time in HCL compared to those who used pump therapy at baseline (Supplementary Table S2). Ensuring that a diverse patient population is represented by study participants is critical and improves generalizability of study findings to the larger population of those living with T1D. Indeed, recent T1DX registry data show that nearly 40% of registry participants are using MDI as their insulin delivery modality, highlighting the need to study this population when designing automated insulin delivery systems.^1^

The cohorts recruited for this study provided a representation of the unique challenges present at various life stages for those living with T1D. The adult cohort entered the study with a mean screening HbA1c only slightly above the target of <7% per ADA standards and a GMI during ST of 6.9%, which is below this target,^24^ but with their usual care regimen the group had a high percentage of time in hypoglycemia. In this generally well-controlled group, the algorithm was able to improve time in target range (70–180 mg/dL) by 5.7% (equivalent to 1.4 h) per day compared to ST, while also significantly reducing time in hypoglycemia <70 mg/dL to below 2%, a reduction from 73 to 27 min each day. This percent time in hypoglycemia falls well below the recommendations for clinical targets recently set by an International Consensus Group^28^ of 4% (58 min). This improvement was achieved while maintaining the mean glucose concentration at 150 mg/dL.

In comparison, the majority of the cohorts of adolescents and children in this study entered with suboptimal screening HbA1c levels per the ISPAD recommended target of <7%,^29,30^ with their percent time in hyperglycemia >180 mg/dL during ST well above recent recommendations for sensor glucose targets.^28^ In particular, children spent nearly half of the overnight hours during ST with sensor glucose readings >180 mg/dL. Parents of children are often fearful of hypoglycemia and may permit hyperglycemia to allay worries of dangerous low glucose levels, especially overnight.^31,32^ Parental fear may be precluding these children from achieving target glucose levels. With recent reports suggesting hyperglycemia in childhood can impact brain development, strategies to mitigate hyperglycemia are essential.^33^ Previous research has shown HCL systems achieve the greatest time in range overnight,^34^ when algorithms do not need to contend with food intake and physical activity. Results of the current trial demonstrated marked reductions in hyperglycemia overnight for children, without increasing hypoglycemia. The Omnipod personalized MPC algorithm was able to significantly increase the time in target range per day by 4.4 h (264 min) for adolescents and 3.4 h (204 min) for children. As these groups had relatively low percent time <70 mg/dL at baseline, there is a potential concern that the use of an HCL system could result in an inadvertent increase in hypoglycemia. However, the mean percent time spent in hypoglycemia actually decreased during HCL, with a significant decrease for adolescents overnight. It is foreseeable that HCL systems have potential to reduce parental fears of hypoglycemia overnight, thus preventing hyperglycemia permissive behaviors.

Although the study was supervised, the algorithm was challenged with real-life scenarios, including unrestricted meals and daily exercise, which provided a relatively unconstrained and representative assessment of the algorithm's performance. By allowing participants to choose meals high in CHOs and fat, we were able to demonstrate that the algorithm could significantly reduce time in hyperglycemia even under these challenging conditions, including 17%–27% of study meals considered high-CHO (≥75 g of CHO) and 21%–68% of meals considered high-fat (≥30 g of fat for adults and adolescents or ≥15 g of fat for children). Physical activity is also a major challenge for anyone requiring insulin therapy as it has the potential to lead to hypoglycemia; however, it also has substantial health benefits.^35^ Adults with T1D are recommended to engage in ≥150 min of moderate intensity aerobic activity per week, and children and adolescents are recommended to engage in ≥60 min per day of moderate-intensity aerobic activity.^36^ Our study included daily physical activity mirroring these recommendations, which provides valuable information to support the safe use of the system by patients across age groups.

Our results are consistent with previous studies, which have shown that HCL systems can be expected to achieve time in target range (70–180 mg/dL) of >70% and time <70 mg/dL of <4% for adults and adolescents,^28,34,37,38^ and time in target range of >65% and time <70 mg/dL of ≤3% for children.^13,21,28^ The results achieved for time with sensor glucose in and below the target range are also consistent with recommended clinical targets for these glycemic measures for adults and youth, which were informed by the results achieved by HCL systems to date.^28^ The relatively short duration of the present study precluded measurement of changes in HbA1c; however, using the GMI,^27^ it is possible to derive participants' estimated HbA1c based on the mean glucose concentration calculated from sensor data from the ST and HCL phases. While GMI is generally suggested to be calculated from at least 10 days of sensor use,^27^ a recent study indicates that 5 days of sensor data can provide an estimation of mean glucose concentration that is reasonably well-correlated with that calculated from a 3-month period of use.^39^ The GMI was an average of 6.9%, 7.2%, and 7.5% based on ST data for adults, adolescents, and children, respectively, compared to an average of 6.9%, 6.8%, and 7.0% based on HCL data. While long-term studies are needed, these results, in combination with the decreased percentage of time in hypoglycemia and hyperglycemia and decreased coefficient of variation during HCL compared to ST, are encouraging and indicate that the Omnipod personalized MPC algorithm has the potential to improve overall glycemic control. Insights gained from the results of the present study were used to further enhance the algorithm to prepare for the next phase of studies.

A strength of this study is that it was designed to be broadly inclusive and represent the larger population living with T1D; therefore, participants were from a wide age spectrum and could be using any baseline insulin delivery modality (MDI or pump). While the relatively short duration of HCL conducted in a supervised hotel/rental home setting may be a limitation, it sets the stage for longer, pivotal trials of the algorithm. Importantly, despite being conducted in a transitional environment, the study mirrored many of the challenges faced in the unsupervised home environment—namely meals of varying size and nutrient composition as well as physical activity sessions of varying intensity, with no protocolization of exercise or meal starting times or durations. Yet, additional challenges to the algorithm may be faced in an unsupervised environment or when the system is used for longer periods of time. Although the 7-day at-home ST phase provided a valuable benchmark to compare glycemic outcomes during HCL to those with the participants' usual therapy, the difference in conditions between the home setting (ST phase) and supervised hotel/rental home setting (HCL phase) means that the ST phase cannot be considered as a true control arm. In addition, participants were not requested to track their meal content or activity during the ST phase, thus limiting the analysis possible between the two study conditions. The adaptivity scheme was included during HCL as a proof-of-concept; however, the study was not designed to determine the effectiveness of this approach. Longer studies with more participants would be needed to determine whether this adaptivity has an effect on glycemic outcomes.

## Conclusions

This feasibility study demonstrated that the Omnipod personalized MPC algorithm performed well and was safe over 96 h of use by adults, adolescents, and children under supervised free-living conditions in an outpatient setting. The algorithm increased the mean time in target range compared to ST, while also decreasing time in hypoglycemia. Indeed, the duration of time spent in target range increased by 5.7%–18.4% (1.4–4.4 h/day) in the various study cohorts. Additional studies are underway to assess the system use in children as young as 2 years old. Long-term outpatient studies will assess safety and performance of the algorithm during extended use in the unsupervised, home environment in patients of all ages with T1D regardless of insulin delivery modality at study entry.

## Supplementary Material

Supplemental data

Supplemental data
