# Supplementary material for: Safety and Performance of the Omnipod Hybrid Closed-Loop System in Adults, Adolescents, and Children with Type 1 Diabetes Over 5 Days Under Free-Living Conditions
Source: Diabetes Technol Ther. 2020 Feb 21;22(3):174–84. doi: 10.1089/dia.2019.0286 (PMC7047109; doi:10.1089/dia.2019.0286)
Supplement: Supplemental data [file Supp_TableS1.pdf]

Supplementary Data

Supplementary Table S1. Glycemic Outcomes for Participants with Adjustments to Pump Settings After the First 48 h of Hybrid Closed-Loop

	Adults (N = *1*)		*Adolescents* (N = *6*)		*Children* (N = *5*)		*All cohorts pooled* (N = *12*)	
	Before adjustment	*After adjustment*	*Before adjustment*	*After adjustment*	*Before adjustment*	*After adjustment*	*Before adjustment*	*After adjustment*
Overall								
Mean glucose, mg/dL	154	154	140 ± 22	154 ± 27	171 ± 35	171 ± 26	154 ± 30	161 ± 25
Coefficient of variation, %	19	32	34	33	40	38	38	36
Time in glucose range, %								
<54 mg/dL	0.0	0.0	0.4 ± 0.5	0.1 ± 0.2	0.5 ± 0.5	0.2 ± 0.3	0.4 ± 0.5	0.1 ± 0.2
<70 mg/dL	0.0	1.4	2.8 ± 2.5	1.7 ± 1.6	2.1 ± 1.2	1.2 ± 1.0	2.3 ± 2.0	1.4 ± 1.3
70–180 mg/dL	79.4	70.1	81.7 ± 14.6	73.3 ± 18.2	61.4 ± 20.4	62.4 ± 19.0	73.0 ± 18.8	68.5 ± 17.6
≥250 mg/dL	0.0	5.9	3.9 ± 4.8	4.5 ± 7.0	15.5 ± 14.2	15.0 ± 11.2	8.4 ± 11.1	9.0 ± 9.8

Data are mean ± SD.
SD, standard deviation.
