# Supplementary material for: Safety and Performance of the Omnipod Hybrid Closed-Loop System in Adults, Adolescents, and Children with Type 1 Diabetes Over 5 Days Under Free-Living Conditions
Source: Diabetes Technol Ther. 2020 Feb 21;22(3):174–84. doi: 10.1089/dia.2019.0286 (PMC7047109; doi:10.1089/dia.2019.0286)
Supplement: Supplemental data [file Supp_TableS2.pdf]

Supplementary Table S2. Glycemic Outcomes During Hybrid Closed-Loop Stratified by Standard Therapy Modality (Pump or Multiple Daily Injections)

	Adults		*Adolescents*		*Children*	
	Pump (n = *8*)	*MDI* (n = *3*)	*Pump* (n = *7*)	*MDI* (n = *3*)	*Pump* (n = *12*)	*MDI* (n = *3*)
Percentage time in range, %						
<54 mg/dL	0.2 ± 0.3	0.2 ± 0.3	0.4 ± 0.5	0.2 ± 0.2	0.4 ± 0.4	0.2 ± 0.0
	0.0 [0.0–0.4]	0.0 [0.0–0.5]	0.2 [0.1–0.7]	0.3 [0.0–0.4]	0.3 [0.0–0.7]	0.2 [0.2–0.2]
<70 mg/dL	2.0 ± 1.4	1.4 ± 1.1	2.7 ± 2.0	2.1 ± 2.1	2.2 ± 2.1	2.0 ± 0.8
	1.9 [1.0–3.1]	0.8 [0.6–2.6]	2.6 [0.8–3.0]	1.7 [0.2–4.3]	1.6 [0.6–3.4]	1.9 [1.2–2.8]
70–180 mg/dL	74.7 ± 8.5	71 ± 3.7	79.5 ± 11.2	77.8 ± 18.3	66.9 ± 13.9	78.4 ± 7.3
≥250 mg/dL	3.7 ± 4.1	6.7 ± 4.6	3.5 ± 5.4	3.5 ± 5.1	10.0 ± 9.3	3.0 ± 1.9
	2.7 [1.4–3.7]	5.7 [2.8–11.7]	0.2 [0.0–8.3]	1.2 [0.0–9.3]	6.4 [4.5–13.3]	3.9 [0.9–4.2]
Time in HCL (%)	98.4 ± 1.1	95.4 ± 1.5	98.9 ± 0.7	97.6 ± 2.1	98.2 ± 1.7	98.9 ± 0.5
Hypoglycemic events per participant per day[a]	1.1	0.7	1.1	0.9	1.1	0.8

Data are mean ± SD or median [IQR].

[a]Capillary BG <70 mg/dL.

BG, blood glucose; HCL. hybrid closed-loop; IQR, interquartile range; MDI, multiple daily injections.
